# Influence of Commonly Used Primer Systems on Automated Ribosomal Intergenic Spacer Analysis of Bacterial Communities in Environmental Samples

**DOI:** 10.1371/journal.pone.0118967

**Published:** 2015-03-06

**Authors:** Witoon Purahong, Barbara Stempfhuber, Guillaume Lentendu, Davide Francioli, Thomas Reitz, François Buscot, Michael Schloter, Dirk Krüger

**Affiliations:** 1 UFZ-Helmholtz Centre for Environmental Research, Department of Soil Ecology, Halle (Saale), Germany; 2 Technical University of Munich, Chair for Soil Science, Oberschleissheim, Germany; 3 Helmholtz Zentrum München, Research Unit for Environmental Genomics, Oberschleissheim, Germany; 4 University of Leipzig, Institute of Biology, Leipzig, Germany; 5 German Centre for Integrative Biodiversity Research (iDiv), Leipzig, Germany; Free University of Bozen/Bolzano, ITALY

## Abstract

Due to the high diversity of bacteria in many ecosystems, their slow generation times, specific but mostly unknown nutrient requirements and syntrophic interactions, isolation based approaches in microbial ecology mostly fail to describe microbial community structure. Thus, cultivation independent techniques, which rely on directly extracted nucleic acids from the environment, are a well-used alternative. For example, bacterial automated ribosomal intergenic spacer analysis (B-ARISA) is one of the widely used methods for fingerprinting bacterial communities after PCR-based amplification of selected regions of the operon coding for rRNA genes using community DNA. However, B-ARISA alone does not provide any taxonomic information and the results may be severely biased in relation to the primer set selection. Furthermore, amplified DNA stemming from mitochondrial or chloroplast templates might strongly bias the obtained fingerprints. In this study, we determined the applicability of three different B-ARISA primer sets to the study of bacterial communities. The results from *in silico* analysis harnessing publicly available sequence databases showed that all three primer sets tested are specific to bacteria but only two primers sets assure high bacterial taxa coverage (1406f/23Sr and ITSF/ITSReub). Considering the study of bacteria in a plant interface, the primer set ITSF/ITSReub was found to amplify (*in silico*) sequences of some important crop species such as *Sorghum bicolor* and *Zea mays*. Bacterial genera and plant species potentially amplified by different primer sets are given. These data were confirmed when DNA extracted from soil and plant samples were analyzed. The presented information could be useful when interpreting existing B-ARISA results and planning B-ARISA experiments, especially when plant DNA can be expected.

## Introduction

Bacterial automated ribosomal intergenic spacer analysis (B-ARISA) is a widely used, culture-independent, molecular technique for analyzing bacterial diversity and community structure in various types of habitats, including both terrestrial and aquatic ecosystems [1–5]. B-ARISA is a PCR-based method that estimates the number of bacterial operational taxonomic units (OTUs) based on the length heterogeneity of the 16S-23S ribosomal intergenic spacer region (IGS) [1], [2]. This method is highly sensitive, reliable and reproducible [3, 4]. Considering the length of the bacterial IGS region (100–1500 bps), B-ARISA can potentially discriminate at least 700 bacterial OTUs (using a 2 bp window for binning), so this method may be suitable to use for a large number of samples collected over a range of locations and at different times [4]. However, B-ARISA alone does not provide any taxonomic information and the results may be severely biased in relation to the primer set selection [2].

Thus, the aim of this study was to compare the coverage and specificity of three primer sets *in silico* and *in vitro*, mainly to investigate their applicability for studies of bacterial communities at the plant–soil interface: 1406f/23Sr [1], ITSF/ITSReub [2] and S-D-Bact-1522-b-S-20/L-D-Bact-132-a-A-18 [6]. We used the updated databases from December 2012 to March 2014 and improved B-ARISA PCR conditions [7, 8]. In addition, we evaluated the primer sets in a more meaningful way by examining both forward and reverse primers together (with 1 to 3 mismatches) instead of evaluating each primer separately. Furthermore, we evaluated, for the first time the specificity of these three B-ARISA primer sets to bacteria. To this end, we also tested whether the primer sets would amplify (*in silico*) chloroplast, mitochondrial, fungal, plant and invertebrate sequences.

## Materials and Methods

### Ethics Statement

Field work permits were issued by the responsible environmental offices of the state of Baden-Württemberg, Germany (according to § 72 BbgNatSchG).

### *In silico* testing

To determine the most valuable primer set for the B-ARISA technique, ecoPCR software (http://www.grenoble.prabi.fr/trac/ecoPCR) [9, 10] was used for theoretical sequence amplification by virtual PCR using data from four sets of databases (S1 Databases, S1 Table, S2 Table, S3 Table, S4 Table). The two primer sets (1406f/23Sr and ITSF/ITSReub) that produced the best results from ecoPCR were evaluated further for their coverage and specificity to bacteria using the FastM and ModelInspector tool, implemented in the Genomatix software suite (http://www.genomatix.de/solutions/genomatix-software-suite.html). Some archaeal sequences were also contained in some databases.

### *In vitro* testing

Ten soil samples were obtained from a long-term soil fertilization experiment that has been running for 110 years in Bad Lauchstädt, Germany [11], where different levels of fertilizer application have been compared. Furthermore, wood samples were taken from 10 different logs of two tree species in the Schwäbische Alb Biodiversity Exploratory (five samples from European beech, *Fagus sylvatica* and five samples from Norway spruce, *Picea abies*) [12]. DNA extracts from all samples were processed with B-ARISA as described by Cardinale et al. [2] for primer set ITSF/ITSReub, and as described by Borneman and Triplett [1] modified according to Yannarell et al. [7] and Frossard et al. [8] for primer set 1406f/23Sr (for more details about the material, methods and statistical analysis, see S1 Methods).

## Results

### Coverage and specificity of primer sets revealed by the ecoPCR software

**Fig 1:**
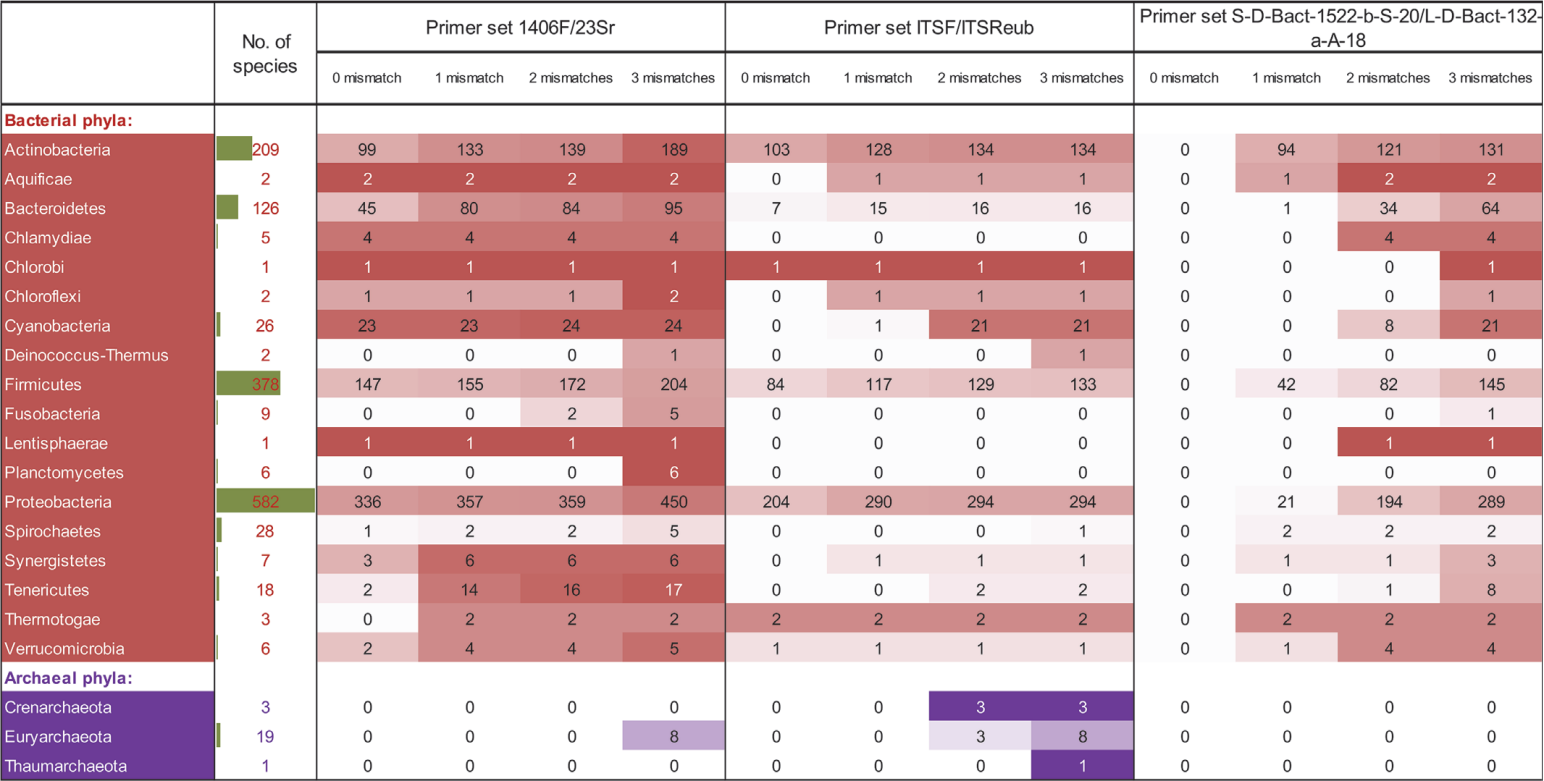
Number of potentially amplified species for different prokaryotic phyla in the wgs-embl-pro database revealed by ecoPCR by increasing mismatches allowed on both forward and reverse primers. The overlaid heatmap (white = 0, darkest = maximum number of sequences) illustrates rising anticipated amplification success with increasing mismatches and was applied per row (i.e. heatmap is proportional to the sequence number available per phylum). The in-cell bar illustrates the relative contribution of the phyla in the used database.

Proportions of bacterial taxa virtually amplified by different primer sets for different levels of bacterial taxonomic classification (from phylum to species) in the prokaryote Whole Genome Sequences database (wgs-embl-pro) retrieved from EMBL are presented in Table 1. Primer set 1406f/23Sr achieved a much higher proportion of bacterial taxa virtually amplified than the other two primer sets at all levels of taxonomic classification when either zero or one mismatch was allowed. For Genome Sequence Scan, High Throughput Genome Sequencing and Standard sequence classes of prokaryotes retrieved from the EMBL (embl-pro) and constrained bacterial 16S-23S spacer (ncbi-bact-spacer) databases, the 1406f/23Sr and ITSF/ITSReub primer sets amplified similar percentages of the bacterial species (zero and one mismatch), with proportions much higher than achieved using the S-D-Bact-1522-b-S-20/L-D-Bact-132-a-A-18 primer set (S1 Table and S2 Table). Fig. 1 shows the total number of bacterial taxa virtually amplified by each primer set allowing zero to three mismatches on both forward and reverse primers from the wgs-embl-pro database. The total numbers of bacterial species or sequences with a positive virtual amplification (separated by phylum) for each primer set from the embl-pro, ncbi-bact-spacer and wgs-embl-pro databases are shown in S1 Table, S2 Table, S3 Table. Overall, the 1406f/23Sr and ITSF/ITSReub primer sets perform better than the S-D-Bact-1522-b-S-20/L-D-Bact-132-a-A-18 primer set by amplifying more bacterial species and sequences in all databases especially when zero and one mismatches was allowed.

**Table 1:** Proportion of bacterial taxa with positive virtual amplification in the wgs-embl-pro database revealed by ecoPCR.

Taxonomic rank	No. of taxa	Primer set 1406F/23Sr		Primer set ITSF/ITSReub		Primer set S-D-Bact-1522-b-S-20/L-D-Bact-132-a-A-18	
		0 mismatch	1 mismatch	0 mismatch	1 mismatch	0 mismatch	1 mismatch
phylum	19	84.2	89.5	36.8	68.4	0	52.6
class	32	78.1	87.5	43.8	59.4	0	46.9
order	72	83.3	91.7	44.4	63.9	0	31.9
family	163	79.8	87.1	49.7	64.4	0	34.4
genus	483	65.4	72.7	33.3	48.9	0	16.8
species	1389	48.2	56.7	28.9	40.3	0	12.0

We tested whether the three primer sets are able to amplify *in silico* the non-target sequences of chloroplast (ncbi-chloro), mitochondria (ncbi-mito), fungi (embl-fun), plant (embl-pln) and invertebrates (embl-inv) (S4 Table). The S-D-Bact-1522-b-S-20/L-D-Bact-132-a-A-18 primer set was most specific for bacterial sequences, only amplifying the IGS region from 4 out of 4945 and 5 out of 115186 species represented in the embl-pln database, respectively for Chlorophyta and Streptophyta. This primer set was unlikely to amplify any chloroplast, mitochondrial, fungal or invertebrate sequences when zero to three mismatches were allowed, except for chloroplast sequences where only one species of Chlorophyta was virtually amplified when 3 mismatches were allowed. The 1406f/23Sr and ITSF/ITSReub primer sets were also specific to bacterial sequences with zero or one mismatch. Primer set 1406f/23Sr virtually amplified 5 out of 27 Chlorophyta (ncbi-chloro), 1 out of 2 Chromerida (ncbi-chloro), 2 out of 5 Euglenida (ncbi-chloro), 16 out of 4945 Chlorophyta (embl-pln) and 2 out of 115186 Streptophyta (embl-pln) species. Primer set ITSF/ITSReub amplified only 8 out of 115186 Streptophyta (embl-pln) and 1 out of 1339 Echinodermata (embl-inv) species. The plant sequences amplified *in silico* by the primer sets 1406f/23Sr and ITSF/ITSReub with zero and one mismatch are presented in S1 Sequences and most of them match with bacterial sequences when blasted against GenBank. When three mismatches were allowed, the 1406f/23Sr and ITSF/ITSReub primer sets amplified *in silico* more chloroplast, fungal and plant species; in addition, ITSF/ITSReub also amplified more invertebrate species. Nevertheless, the proportions of non-bacterial species amplifiable by these two primer sets were very low even when three mismatches were allowed.

### Coverage and specificity of primer sets revealed by the Genomatix software suite

The total number of virtually amplified sequences using each primer set and the number of different genera to which these sequences belong was analyzed. Genera have been grouped into the corresponding bacterial phyla. Representatives of the phyla Chloroflexi, Deinococcus-Thermus, Gemmatimonades and Planctomycetes were only covered by primer set 1406f/23Sr (S5 Table). Primer set ITSF/ITSReub, however, seems not to amplify sequences belonging to any representative of these phyla. The number of sequences and of genera within each phylum varied between the primer sets tested. A table containing a detailed list of the genera included in the analysis can be found in S6 Table and S7 Table. Twenty one amplifiable sequences were found with primer set1406f/23Sr and 12 sequences with ITSF/ITSReub in the Genomatix database for plant-assigned sequences (S5 Table). Primer set 1406f/23Sr resulted in sequence hits for green algae (Chlorophyta) to a large extent, whilst red algae (Rhodophyta) and sequences of genus *Zea* were only found for the set ITSF/ITSReub. *Paulinella*, belonging to Rhizaria, could be amplified by both primer sets. In both cases, most sequences were identified as plastid / chloroplast sequences. Besides a few sequences without known genus and not reported in the result tables, the ITS1F/ITSReub primer pair could amplify *Methanocella* of the domain Archaea.

### *In vitro* testing

**Fig 2:**
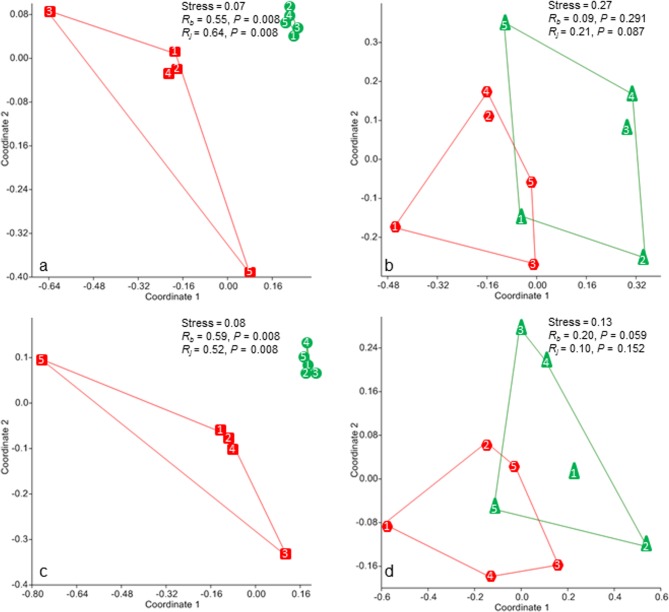
NMDS ordination plots of bacterial community structure in soil (a, c) and wood (b, d) samples using different primer sets: 1406f/23Sr (a, b) and ITSF/ITSReub (c, d). Stress values from the NMDS ordinations and *R*_*ANOSIM*_ based on Bray-Curtis (*R*_*b*_) and Jaccard (*R*_*j*_) distance measures are shown on the right. Square = fertilized soil, circle = unfertilized soil, hexagon = *Picea abies*, triangle = *Fagus sylvatica*. Each number (1 to 5) represents one individual replicate.

The figures for bacterial richness and community structure obtained using the 1406f/23Sr and ITSF/ITSReub primer sets were similar (S8 Table; Fig. 2). The correlation between the two primer sets with respect to OTU richness and Shannon diversity was significant for plant samples (OTU richness: *r* = 0.67, *P* = 0.03; Shannon diversity *r* = 0.89, *P* = 0.0007) but not for soil samples (OTU richness: *r* = −0.10, *P* = 0.43; Shannon diversity *r* = 0.24, *P* = 0.46). When we examined the results for each primer set in order to determine the effects of fertilization (unfertilized *vs*. fertilized soil) and tree species (Norway spruce *vs*. European beech) on bacterial richness and community structure, similar results were obtained regardless of the primer set used.

## Discussion and Conclusions

Under changing conditions attributed to the rapid database expansions and new software tools for analyzing the specificity of primer systems, in our study we could show that the 1406f/23Sr and ITSF/ITSReub can be considered as the most promising primer sets for B-ARISA. However those results have to be interpreted in the light of the paucity of the publicly available sequence databases. The wgs-embl-pro was used as the most accurate database to estimate the amplification potential of the IGS region as all tested species were supposed to have the same chance to be virtually amplified by the different primer sets. This is equally true for the non-target ncbi-chloro and ncbi-mito databases as they contain full genomes of chloroplast and mitochondria, respectively. For the other databases however, most of the sequences do not cover the IGS region (embl-pro, -pln, -fun, -inv) or do not contain the region targeted by the primers (ncbi-bact-spacer) resulting in low virtual amplification rates, even for the targeted prokaryotic phyla (S1 Table and S2 Table).

For the primer set ITSF/ITSReub, we could confirm its high coverage and specificity for bacteria when 0 to 1 mismatch was allowed, as obtained by Cardinale et al. [2]. However, for the primer set 1406F/23Sr, current bioinformatics as well as advances in analytical methodology reveal contrasting results compared to a similar study carried out on a datasets almost a decade older [2]. An improvement in the soil DNA extraction method and/or different PCR conditions we used for our B-ARISA for the 1406f/23Sr primer set could also have increased the quality of the B-ARISA fingerprints obtained. Cardinale et al. [2] reported that with the 1406f/23Sr primer set no B-ARISA peak from soil samples (including natural and polluted soil) could be obtained. However, in our study we found that the 1406f/23Sr primer set was quite able to amplify bacterial DNA templates in natural and fertilized soil and the numbers of B-ARISA peaks (OTUs) obtained by 1406f/23Sr and ITSF/ITSReub were not significantly different. When we examined the results for each primer set in order to determine the effects of fertilization (unfertilized vs fertilized soil) and tree species (Norway spruce *vs*. European beech) on bacterial richness and community structure, similar results were obtained regardless of the primer set used. We could also show that, if primers used have comparable properties based on *in silico* analysis, the data obtained for diversity and richness of bacterial communities based on ARISA were highly similar, independent of the studied habitat, which has also been postulated by others [4]. However, we suggest that the bias of each primer set should be taken into consideration when selecting a suitable primer set for each particular experiment. We list bacterial genera and plant species potentially amplified by primer sets 1406F-23Sr and ITSF/ITSFReub; this information could be useful when interpreting existing B-ARISA results and planning B-ARISA experiments involving samples containing plant material.

In conclusion, we consider that B-ARISA is still a powerful tool for analyzing bacterial communities, especially for simple communities originating from a restricted area or a controlled system with known bacterial community composition and biases. Using B-ARISA to investigate complex bacterial communities may still be valuable as it can provide a quick snapshot of bacterial richness and community composition before applying more sensitive approaches such as amplicon sequencing. The usefulness of B-ARISA patterns can also be seen in the study of Gobet et al. [13] where they were ecologically coherent with the data obtained from 454 pyrosequencing.

## Supporting Information

S1 Table Number of species with positive virtual amplification for different prokaryotic phyla in the embl-pro database (41 phyla, 1200281 sequences) revealed by ecoPCR. (XLSX)

S2 Table Number of species with positive virtual amplification for different bacterial phyla in ncbi-bac-spacer database (19 phyla, 37134 sequences) revealed by ecoPCR. (XLSX)

S3 Table Number of amplified sequences for different prokaryotic phyla in wgs-embl-pro database (21 phyla, 178462 sequences) revealed by ecoPCR. (XLSX)

S4 Table Non-target species amplifiable by the different primer sets. Table shows the potential ability of primer sets to amplify mitochondrial, chloroplast, fungal, plant and invertebrate sequences.
(XLSX)

S5 Table Number of amplifiable sequences from different bacterial, archaeal, plant and invertebrate phyla and genera revealed by Genomatix software suite. (XLSX)

S6 Table List of bacterial genera included for Genomatix analysis. (XLS)

S7 Table Bacterial genera potentially amplified by primer sets 1406f/23Sr (a) and ITSF/ITSFReub (b). (XLSX)

S8 Table B-ARISA analyses in soil and wood samples for 1406f/23Sr and ITSF/ITSReub primer sets. (XLS)

S1 Databases (DOC)

S1 Methods (DOC)

S1 Sequences (DOC)
