# Supplementary material for: Influence of Commonly Used Primer Systems on Automated Ribosomal Intergenic Spacer Analysis of Bacterial Communities in Environmental Samples
Source: PLoS One. 2015 Mar 6;10(3):e0118967. doi: 10.1371/journal.pone.0118967 (PMC4351999; doi:10.1371/journal.pone.0118967)
Supplement: S1 Databases — (DOC) [file pone.0118967.s009.doc]

Influence of commonly used primer systems on automated ribosomal intergenic spacer analysis of bacterial communities in environmental samples

Witoon Purahong, Barbara Stempfhuber, Guillaume Lentendu, Davide Francioli, Thomas Reitz, François Buscot, Michael Schloter, Dirk Krüger

**S1 Databases**

ecoPCR analyses were performed on four sets retrieved from databases to test coverage and specificity of different B-ARISA primer sets. Though the plant databases actually include other groups of organisms such as algae and protozoa, we simply used the term “plant” to refer to the original name of each database.

First, all Genome Sequence Scan, High Throughput Genome Sequencing and Standard sequence classes of fungi, invertebrates, plants and prokaryotes were retrieved from the EMBL release 118 of December 2013 (embl-fun, embl-inv, embl-pln, embl-pro).

Second, Whole Genome Sequences of prokaryotes were retrieved from the EMBL release 114 of December 2012, and only one entry per species name was conserved for further analysis (wgs-embl-pro).

Third, a more constrained database was retrieved from the NCBI GenBank nucleotide database using the key search “((16S-23S spacer) AND "bacteria"[porgn:__txid2]) AND 200:1000000[Sequence Length]” on March 2013 (Release 194, ncbi-bact-spacer) in order to restrict the database to the potential target sequences (i.e. bacterial intergenic spacer) of the analysed primer pairs.

Finally, the available full chloroplast and mitochondrial genomes were retrieved from the NCBI Organelle Genome Ressources on March 2014 (ncbi-chloro and ncbi-mito).
