# Supplementary material for: Influence of Commonly Used Primer Systems on Automated Ribosomal Intergenic Spacer Analysis of Bacterial Communities in Environmental Samples
Source: PLoS One. 2015 Mar 6;10(3):e0118967. doi: 10.1371/journal.pone.0118967 (PMC4351999; doi:10.1371/journal.pone.0118967)
Supplement: S1 Methods — (DOC) [file pone.0118967.s010.doc]

Influence of commonly used primer systems on automated ribosomal intergenic spacer analysis of bacterial communities in environmental samples

Witoon Purahong, Barbara Stempfhuber, Guillaume Lentendu, Davide Francioli, Thomas Reitz, François Buscot, Michael Schloter, Dirk Krüger

**S1 Methods**

**Material and methods**

Soil and plant samples

Ten soil samples were obtained from the Static Fertilization Experiment in Bad Lauchstädt (Northeastern Germany), a long-term soil fertilization experiment running for over 110 years, (Merbach and Schulz 2012). Five soil samples belong to control (unfertilized soil) and the rest belongs to samples that were treated with manure and chemical fertilized soil (20 t/ha). In addition, ten wood samples were taken from ten different logs of two different tree species (5 samples from European beech, *Fagus sylvatica* and another 5 samples from Norway spruce, *Picea abies*), all from the Schwäbische Alb Biodiversity Exploratory (Fischer et al., 2010). The logs from these two tree species were comparable in length, diameter and decay class. The samples taken from the same log were combined, homogenized, ground into a fine powder with the aid of liquid nitrogen using a swing mill (Retsch, Haan, Germany) and stored at -80 ºC.

DNA extraction and B-ARISA

DNA was extracted from 250 mg homogenized soil or 100 mg homogenized wood samples, using the ZR Soil Microbe DNA MiniPrep kit (Zymo Research, Irvine, CA, USA), according to the manufacturer’s instructions. The presence and quantity of genomic DNA was checked using a NanoDrop ND-1000 spectrophotometer (Thermo Fisher Scientific, Dreieich, Germany) and the extracts were then stored at -20ºC. B-ARISA was performed in duplicate reactions using 1µl DNA template solution (20 ng template as determined by NanoDrop) under the conditions described by Cardinale et al. (2004) (primer set ITSF/ITSReub) and Borneman and Triplett (1997) modified according to Frossardet al. (2012) (primer set 1406f/23Sr). For the primer set 1406f/23Sr, briefly, the PCR mixture (20 μl) contained 1 μl DNA template (~20 ng DNA template as determined by NanoDrop); 10 μM of primer 1406f (5′-TGYACACACCGCCCGT-3′) labeled with FAM at 5′-end and an unlabeled 23Sr primer (5′-GGGTTBCCCCATTCRG-3′); 4 μl FIREPol 5x Master Mix (Solis BioDyne, Tartu, Estonia); and water to 20 µl. PCR was carried out with an initial denaturation at 94 °C for 5 min, followed by 30 cycles of 94 °C for 35 s, 55 °C for 45 s and 72 °C for 2 min, with a final extension at 72 °C for 5 min. The PCR products were purified using a PCRExtract Mini Kit (5PRIME, Hamburg, Germany). A standardized quantity of DNA (40 ng of DNA, as determined by NanoDrop) was mixed with 14 µl of deionized Hi-Di formamide (Applied Biosystems, Foster City, CA, USA) and 0.1 µl of internal size standard Map Marker 1500 ROX (50-1500 bp) (BioVentures, Inc, Murfreesboro, TN, USA). The mixture was denatured for 5 min at 95^o^C and chilled on ice for at least 10 min before being further processed on a capillary sequencer (ABI PRISM 3730xl Genetic Analyzer, Applied Biosystems). The two independent PCR replicates were highly correlated (ρ = 0.84, *P* < 0.0001; data not shown). All peaks of the fragments between 200 and 1500 bp that appeared in two technical PCR replicates were used for further analyses (Frossardet al., 2012). Operational taxonomic unit (OTU) binning was carried out using an interactive custom binning script (Ramette, 2009) in R version 2.14.1 (The R Foundation for Statistical Computing, 2011-2012) (binning size = 2 bp, cut off = 0.09%). Double DNA normalization steps before the initial PCR and the separation of DNA fragments via capillary electrophoresis make this standard F-ARISA robust for inferring changes in community structure (Ramette, 2009).

Primer pairs targeting the bacterial 16S-23S intergenic spacer

|  | Forward | Reverse | Reference |
| --- | --- | --- | --- |
| 1406f/23Sr | TGYACACACCGCCCGT | GGGTTBCCCCATTCRG | Borneman and Triplett, 1997 |
| ITSF/ITSReub | GTCGTAACAAGGTAGCCGTA | GCCAAGGCATCCACC | Cardinale et al., 2004 |
| S-D-Bact-1522-b-S-20/L-D-Bact-132-a-A-18 | TGCGGCTGGATCCCCTCCTT | CCGGGTTTCCCCATTCGG | Ranjard et al., 2000 |

**Statistical analysis**

B-ARISA fingerprint data were analyzed using the PAST program (Hammer et al., 2001). OTU richness of different treatments in soil and wood samples were analyzed using the paired t-test incorporating the Jarque-Bera JB test for normality and the *F* test for the equality of group variances, while the bacterial community structures were assessed using one-way ANOSIM based on Bray-Curtis and Jaccard distance measures. Non-metric multidimensional scaling (NMDS) based on Bray-Curtis distance measure was performed to visualize the bacterial community structure of different treatments.

**References**

Borneman, J., Triplett, E.W., 1997. Molecular microbial diversity in soils from eastern Amazonia: evidence for unusual microorganisms and population shifts associated with deforestation. Appl. Environ. Microbiol. 63, 2647– 2653.

Cardinale, M., Brusetti, L., Quatrini, P., Borin, S., Puglia, A. M., Rizzi, A., Zanardini, E., Sorlini, C., Corselli, C., Daffonchio, D., 2004. Comparison of different primer sets for use in automated ribosomal intergenic spacer analysis of complex bacterial communities. Appl. Environ. Microbiol. 70, 6147–6156.

Frossard, A., Gerull, L., Mutz, M., Gessner, M. O., 2012. Fungal importance extends beyond litter decomposition in experimental early-successional streams. Environ. Microbiol. 14, 2971-2983.

Merbach, I., Schulz, E., 2012. Long-term fertilization effects on crop yields, soil fertility and sustainability in the Static Fertilization Experiment Bad Lauchstädt under climatic conditions 2001–2010. Arch. Agron. Soil. Sci. 59, 1041-1057.

Ranjard, L., Brothier, E., Nazaret, S., 2000. Sequencing bands of ribosomal intergenic spacer analysis fingerprints for characterization and micro scale distribution of soil bacterium populations responding to mercury spiking. Appl. Environ. Microbiol. 66, 5334–5339.
