# Supplementary material for: Influence of Commonly Used Primer Systems on Automated Ribosomal Intergenic Spacer Analysis of Bacterial Communities in Environmental Samples
Source: PLoS One. 2015 Mar 6;10(3):e0118967. doi: 10.1371/journal.pone.0118967 (PMC4351999; doi:10.1371/journal.pone.0118967)
Supplement: S1 Sequences — (DOC) [file pone.0118967.s011.doc]

Influence of commonly used primer systems on automated ribosomal intergenic spacer analysis of bacterial communities in environmental samples

Witoon Purahong, Barbara Stempfhuber, Guillaume Lentendu, Davide Francioli, Thomas Reitz, François Buscot, Michael Schloter, Dirk Krüger

**S1 Sequences**

Fragment of the plant sequences *in silico* amplified in the embl-pln databases at 0 and 1 mismatch allowed. Sequences are annotated with their original sequence identifier, their species name, their phylum (if available or “None”) and the primer pair which virtually amplified it.

>FJ789639 *Brassica rapa* Streptophyta 1406f/23Sr

cacaccatgggagttggattcactcgaaggcgttgagctaaccgtaaggaggcaggcgac

cacagtgggtttagcgactggggtgaagtcgtaacaaggtagccgtaggggaacctgcgg

ctggatcacctcctttctaaggatcgtgacgaaagcgtcagtgctagacactgaaagagc

ttcgtcatttccaaagaacatagccgccgtcctcatgtcccttcatcactagagattagc

gcagttcgctgcgctgatagctgagcaggctcaagcgcctctggctgctaacgcagcctg

atttggcagctgggccggtagctcaggtggttagagcgcacgcctgataagcgtgaggtc

ggaggttcaactcctccccggcccaccagcatttggtgaggggctttagctcagctggga

gagcggttgctttgcaagcatcaggtcatcggttcgatcccgataagctccaccatttgc

tgcgcaaatggtcttttgatttgggtccgctgcataagcagcgacggccgttcggccttg

cgaaccttcggttcgttggagcgccaatcaagcgatctgccagatactctagttgatgaa

gatagcggtttaccagccaaggctggtgatatggctcgcacaagcgagcctctttgacat

tgtgaatgggttttttaatcgatgccgtggcgacatggttcggtttttggtgtttccgca

agggagcatccggaaagcgggcgatgttgtacacacaagattatctggctgagtttaata

accacaccgatacagctttcgacaaatgctacccagtattgtcgttggtggtgtggactc

tcaagcgtgaggtaagggcatctggtgaatgccttggcatgtacaggcgatgaaggacgt

ggcacgctgcgataagcgtgggggagccgtgagcaggctttgatcccgcgattt

>GU799579 *Carica papaya* Streptophyta 1406f/23Sr

cacaccatgggagtgggttgcaaaagaagtaggtagcttaaccttcgggagggcgcttac

cactttgtgattcatgactggggtgaagtcgtaacaaggtaaccgtaggggaacctgcgg

ttggatcacctccttaccttaaagaagcgttctttgcagtgctcacacagattgtctgat

agaaagtgaaaagcaaggcgtttacgcgttgggagtgaggctgaagagaataaggccgtt

cgctttctattaatgaaagctcaccctacacgaaaatatcacgcaacgcgtgataagcaa

ttttcgtgtccccttcgtctagaggcccaggacaccgccctttcacggcggtaacagggg

ttcgaatcccctaggggacgccacttgctggtttgtgagtgaaagtcacctgccttaata

tctcaaaactcatcttcgggtgatgtttgagatatttgctctttaaaaatctggatcaag

ctgaaaattgaaacactgaacaacgaaagttgttcgtgagtctctcaaattttcgcaaca

cgatgatgaatcgaaagaaacatcttcgggttgtgaggttaagcgactaagcgtacacgg

tggatgccctggcagtcagaggcgatgaaggacgtgctaatctgcgataagcgtcggtaa

ggtgatatgaaccgttataaccggcgattt

>AF393605 *Pedinomonas minor* Chlorophyta 1406f/23Sr

cacaccacgggagctggctatgcccaaagtcgttaccccaaccgtttggagggggatgcc

taaggcagagctagtgactggggtgaagtcgtaacaaggtagccgtactggaaggtgcgg

ctggatcacctcctttttaaggtcatttaatgacttaaaaaatgcctttggtatttttta

agttcttttaaaactctaagcaaagctcagctccactaagagctggggctattagctcag

gtggttagagcgcacccctgataagggtgaggtcgctggttcgagtccagcatagcccat

ggcttacaaaagcttggagtagtttcataattggtttgtactgggggtatagctcagttg

gtagagcgctgcctttgcaaggcagatgtcagcggttcgagtccgcttacctccaccaaa

tcaaaagtgattacactttttaaaagaataattttttaacgtttttcagctcaattctat

tgagcataaaaacaaatccatggtcaaaggacatacggattacggtggatacctaggcac

ttagagtcgatgaagggcgtggaaaccaacgaaatgcttcggggagctggaaacaagcta

cgatccggagattc

>AM084273 *Paulinella chromatophora* None 1406f/23Sr

cacaccatggaagttggccatgcccgaagtcgttacttcaacccttgtggaggaggacgc

cgaaggtggggctgatgactggggtgaagtcgtaacaaggtagccgtaccggaaggtgcg

gctggatcacctcctaacagggagacaaaatgtgttactaatgtttggctacttatattg

ccattctggtatcctgtcaccttaggtcgatcggtatcttatttcgtaagtctggatttt

gtaattcagctatcatttcagttcctaaactttgtctaagtcatgccccggcaagggtct

cctgggccattagctcaggtggttagagcgcacccctgataagggtgaggtccctggttc

aagtccaggatggcccattcgtgtttgggggtttagctcagttggtagagcgcctgcttt

gcaagcaggatgtcagcggttcaaatccgctaacctccattgaccaaattcttctaaatc

aagaaggaagatctttgaagtagcatgagactagacacctaagctgaattagaattcagc

atttttcttacttagactggtcaaactaaaagtaaaaccagtctaagtaagaaaaatgct

gaactccttgtaatgagactaaatcttagttttcattagagatagttcggccgaaccttg

acaactacataggcaaaattagaagaataagtttcttatgggggcttacctaaataatag

tagtccatttgttagactattgctataaggataagcttctagccagaataaaatttgatg

ttttgatataaatgcctgcttttaatttgattaaaacaggccaaaatcatgatattagtg

aatctattttgatgatctgccctcgtgattttgaagtttatcaaaaaattaatctggtta

agctacaaagagctcacggtggataccttggcacacagaggcgatgaaggacgtggttac

ctgcgataagtctcggggagctggaaacacgctttgatccgggaattt

>CP000815 *Paulinella chromatophora* None 1406f/23Sr

cacaccatggaagttggccatgcccgaagtcgttacttcaacccttgtggaggaggacgc

cgaaggtggggctgatgactggggtgaagtcgtaacaaggtagccgtaccggaaggtgcg

gctggatcacctcctaacagggagacaaaatgtgttactaatgtttggctacttatattg

ccattctggtatcctgtcaccttaggtcgatcggtatcttatttcgtaagtctggatttt

gtaattcagctatcatttcagttcctaaactttgtctaagtcatgccccggcaagggtct

cctgggccattagctcaggtggttagagcgcacccctgataagggtgaggtccctggttc

aagtccaggatggcccattcgtgtttgggggtttagctcagttggtagagcgcctgcttt

gcaagcaggatgtcagcggttcaaatccgctaacctccattgaccaaattcttctaaatc

aagaaggaagatctttgaagtagcatgagactagacacctaagctgaattagaattcagc

atttttcttacttagactggtcaaactaaaagtaaaaccagtctaagtaagaaaaatgct

gaactccttgtaatgagactaaatcttagttttcattagagatagttcggccgaaccttg

acaactacataggcaaaattagaagaataagtttcttatgggggcttacctaaataatag

tagtccatttgttagactattgctataaggataagcttctagccagaataaaatttgatg

ttttgatataaatgcctgcttttaatttgattaaaacaggccaaaatcatgatattagtg

aatctattttgatgatctgccctcgtgattttgaagtttatcaaaaaattaatctggtta

agctacaaagagctcacggtggataccttggcacacagaggcgatgaaggacgtggttac

ctgcgataagtctcggggagctggaaacacgctttgatccgggaattt

>CP000815 *Paulinella chromatophora* None 1406f/23Sr

cacaccatggaagttggccatgcccgaagtcgttacttcaacccttgtggaggaggacgc

cgaaggtggggctgatgactggggtgaagtcgtaacaaggtagccgtaccggaaggtgcg

gctggatcacctcctaacagggagacaaaatgtgttactaatgtttggctacttatattg

ccattctggtatcctgtcaccttaggtcgatcggtatcttatttcgtaagtctggatttt

gtaattcagctatcatttcagttcctaaactttgtctaagtcatgccccggcaagggtct

cctgggccattagctcaggtggttagagcgcacccctgataagggtgaggtccctggttc

aagtccaggatggcccattcgtgtttgggggtttagctcagttggtagagcgcctgcttt

gcaagcaggatgtcagcggttcaaatccgctaacctccattgaccaaattcttctaaatc

aagaaggaagatctttgaagtagcatgagactagacacctaagctgaattagaattcagc

atttttcttacttagactggtcaaactaaaagtaaaaccagtctaagtaagaaaaatgct

gaactccttgtaatgagactaaatcttagttttcattagagatagttcggccgaaccttg

acaactacataggcaaaattagaagaataagtttcttatgggggcttacctaaataatag

tagtccatttgttagactattgctataaggataagcttctagccagaataaaatttgatg

ttttgatataaatgcctgcttttaatttgattaaaacaggccaaaatcatgatattagtg

aatctattttgatgatctgccctcgtgattttgaagtttatcaaaaaattaatctggtta

agctacaaagagctcacggtggataccttggcacacagaggcgatgaaggacgtggttac

ctgcgataagtctcggggagctggaaacacgctttgatccgggaattt

>DQ291132 *Oltmannsiellopsis viridis* Chlorophyta 1406f/23Sr

cacaccatggaagttggttccgcccgaagtcgtggatctaaccttttggaagaaagcgcc

tacggtgtaactagtaactatggtgaagtcgtaacaaggtatccgtactggaaggtgtgg

attggaatacctcctttaaaggacaaaataatgcgtagcgaagtcctttttgactttgct

agtgctttttgtttaacagctacctttgaggtagcctttggctattatgttcctcccccc

gggaaggttcccagggggaggaacataatggttttaatatttgcggtggcgtgaatcgat

attcacggcccaggtccggtttatccgggcacgggtatatagctcagttggtagagcgct

gtctttgcacggcagatgtcagcggttcgaatccgcttatatccacctgaaatatatttc

gagtcgggctattagctcagttggttagagcgcgcccctgataagggcgaggtcgctggt

tcaaatccagcatagcccactcgaatcagtgctgttaagttacagcacatagcatccacc

caattagcttagctagttgggtggggcatccaaagatcaactagctaatttaaagtttgc

tagtggttttgtggtcaaatgacaataggcttatggtggatacctaggcacccaaagttg

atgaagggcgcggaaaccggcgaaacgcttcggggagttggaaacacgctttgatccgaa

gatac

>DQ291132 *Oltmannsiellopsis viridis* Chlorophyta 1406f/23Sr

cacaccatggaagttggttccgcccgaagtcgtggatctaaccttttggaagaaagcgcc

tacggtgtaactagtaactatggtgaagtcgtaacaaggtatccgtactggaaggtgtgg

attggaatacctcctttaaaggacaaaataatgcgtagcgaagtcctttttgactttgct

agtgctttttgtttaacagctacctttgaggtagcctttggctattatgttcctcccccc

gggaaggttcccagggggaggaacataatggttttaatatttgcggtggcgtgaatcgat

attcacggcccaggtccggtttatccgggcacgggtatatagctcagttggtagagcgct

gtctttgcacggcagatgtcagcggttcgaatccgcttatatccacctgaaatatatttc

gagtcgggctattagctcagttggttagagcgcgcccctgataagggcgaggtcgctggt

tcaaatccagcatagcccactcgaatcagtgctgttaagttacagcacatagcatccacc

caattagcttagctagttgggtggggcatccaaagatcaactagctaatttaaagtttgc

tagtggttttgtggtcaaatgacaataggcttatggtggatacctaggcacccaaagttg

atgaagggcgcggaaaccggcgaaacgcttcggggagttggaaacacgctttgatccgaa

gatac

>FJ858267 *Micromonas sp. RCC299* Chlorophyta 1406f/23Sr

cacaccatggaagccgactcttccctaagtcgttactcgaacccattgggacgaggatgc

cgaaggcagggttggtgactggggtgaagtcgtaacaaggtagccgtactggaaggtgtg

gctggaatacctcctttttaaagatgaaacaagcgcgcatttcttaaaacaattggcttg

ccattgcttgagaagaaatgcaccacgctacgggctattagctcaggtggttagagcgca

cccctgataagggtgaggtctcaggttcaagtcctgaatagcccatggcacgggggtata

gctcagttggtagagcgctgcctttgcaaggcagacgccagcggttcgagtccgcttacc

tccagccaagcctttaatttaaaaggtcaattagaattgtggttatttctacacataaaa

caaaatcaatcaataaagggcatatggtggagacctagggacctagagccgatgaagggc

gtgacaaccgacgatatgcctcggggagcaggacgtatgctttgatccgaggattc

>FJ858267 *Micromonas sp. RCC299* Chlorophyta 1406f/23Sr

cacaccatggaagccgactcttccctaagtcgttactcgaacccattgggacgaggatgc

cgaaggcagggttggtgactggggtgaagtcgtaacaaggtagccgtactggaaggtgtg

gctggaatacctcctttttaaagatgaaacaagcgcgcatttcttaaaacaattggcttg

ccattgcttgagaagaaatgcaccacgctacgggctattagctcaggtggttagagcgca

cccctgataagggtgaggtctcaggttcaagtcctgaatagcccatggcacgggggtata

gctcagttggtagagcgctgcctttgcaaggcagacgccagcggttcgagtccgcttacc

tccagccaagcctttaatttaaaaggtcaattagaattgtggttatttctacacataaaa

caaaatcaatcaataaagggcatatggtggagacctagggacctagagccgatgaagggc

gtgacaaccgacgatatgcctcggggagcaggacgtatgctttgatccgaggattc

>FJ968740 *Pedinomonas minor* Chlorophyta 1406f/23Sr

cacaccacgggagctggctatgcccaaagtcgttaccccaaccgtttggagggggatgcc

taaggcagagctagtgactggggtgaagtcgtaacaaggtagccgtactggaaggtgcgg

ctggatcacctcctttttaaggtcatttaatgacttaaaaaatgcctttggtatttttta

agttcttttaaaactctaagcaaagctcagctccactaagagctggggctattagctcag

gtggttagagcgcacccctgataagggtgaggtcgctggttcgagtccagcatagcccat

ggcttacaaaagcttggagtagtttcataattggtttgtactgggggtatagctcagttg

gtagagcgctgcctttgcaaggcagatgtcagcggttcgagtccgcttacctccaccaaa

tcaaaagtgattacactttttaaaagaataattttttaacgtttttcagctcaattctat

tgagcataaaaacaaatccatggtcaaaggacatacggattacggtggatacctaggcac

ttagagtcgatgaagggcgtggaaaccaacgaaatgcttcggggagctggaaacaagcta

cgatccggagattc

>FJ968740 *Pedinomonas minor* Chlorophyta 1406f/23Sr

cacaccacgggagctggctatgcccaaagtcgttaccccaaccgtttggagggggatgcc

taaggcagagctagtgactggggtgaagtcgtaacaaggtagccgtactggaaggtgcgg

ctggatcacctcctttttaaggtcatttaatgacttaaaaaatgcctttggtatttttta

agttcttttaaaactctaagcaaagctcagctccactaagagctggggctattagctcag

gtggttagagcgcacccctgataagggtgaggtcgctggttcgagtccagcatagcccat

ggcttacaaaagcttggagtagtttcataattggtttgtactgggggtatagctcagttg

gtagagcgctgcctttgcaaggcagatgtcagcggttcgagtccgcttacctccaccaaa

tcaaaagtgattacactttttaaaagaataattttttaacgtttttcagctcaattctat

tgagcataaaaacaaatccatggtcaaaggacatacggattacggtggatacctaggcac

ttagagtcgatgaagggcgtggaaaccaacgaaatgcttcggggagctggaaacaagcta

cgatccggagattc

>FN563075 *Oltmannsiellopsis viridis* Chlorophyta 1406f/23Sr

cacaccatggaagttggttccgcccgaagtcgtggatctaaccttttggaagaaagcgcc

tacggtgtaactagtaactatggtgaagtcgtaacaaggtatccgtactggaaggtgtgg

attggaatacctcctttaaaggacaaaataatgcgtagcgaagtcctttttgactttgct

agtgctttttgtttaacagctacctttgaggtagcctttggctattatgttcctcccccc

gggaaggttcccagggggaggaacataatggttttaatatttgcggtggcgtgaatcgat

attcacggcccaggtccggtttatccgggcacgggtatatagctcagttggtagagcgct

gtctttgcacggcagatgtcagcggttcgaatccgcttatatccacctgaaatatatttc

gagtcgggctattagctcagttggttagagcgcgcccctgataagggcgaggtcgctggt

tcaaatccagcatagcccactcgaatcagtgctgttaagttacagcacatagcatccacc

caattagcttagctagttgggtggggcatccaaagatcaactagctaatttaaagtttgc

tagtggttttgtggtcaaatgacaataggcttatggtggatacctaggcacccaaagttg

atgaagggcgcggaaaccggcgaaacgcttcggggagttggaaacacgctttgatccgaa

gatac

>FN563083 *Nephroselmis pyriformis* Chlorophyta 1406f/23Sr

cacaccatggaagctggccatccccgaagtcattaccctaacccattgggaggggggtgc

ctaaggcagggctagtgactagggtgaagtcgtaacaaggtagccgtactggaaggtgcg

gctggatcacctccttttcaggaaaaaaacgcgagcctcgtaggctcgcacgggctatta

gctcagctggttagagcgcacccctgataagggtgaggacgctggttcgagtccagcata

gcccaacgggggtatagctcagttggtagagcgctgcctttgcaaggcagatgtcagcgg

ttcgagtccgcttacctccacaaatttggtcaaatgaataagagcacatggtggatacct

gggcacctagagacgaagaagggcgtcactaccgacgatacgctccggggagctggaagt

aagcttcgatccggagattc

>FN563093 *Crustomastix stigmatica* Chlorophyta 1406f/23Sr

cacaccctgggagttagttttgtccgaagtcgttactctaaccgtaaggaagtggacgcc

gaagtcagagctggcgacctgggtgaagtcgtaacaaggtaaccgtactggaaggtgcgg

ttagaatacctccttctacatgaagtatatgcggagcacacccccgcagacctcccagtc

tcggggtgggtgctcaaacatgggctattagctcaggtggttagagcgcacccctgataa

gggtgaggtccctggttcgagtccaggatagcccacccacctcacgaagggtgtcgattg

gcacccgagggtgggggtatagctcagttggtagagcgcagcctttgcaaggctgatgcc

agcggttcgagtccgcttacctccatagcgagggggtgggagatgaatttttgaagaaga

aggacgcaaagcaggatgaaatgaatattgaaagggcgcatggcggattcctaggcatct

agaggcgatgaagggcgtgactaccgacgaaacgctccagggagccggaaaaaggttttg

atctggagattc

>FN563094 *Dolichomastix tenuilepis* Chlorophyta 1406f/23Sr

cacactatggaagtttactatacccaaagccattaccttaaccttttggagagggatgtc

tacggtagggtcaataactggagtgaagtcgtaacaaggtagcagtactggaaggtgcgg

ctggattacctccttttttaaggataaaattaaatatatataactttgtgaatctttgta

aagattccattatgaggtaaatcaatgataacctcaaaaatctatctaagatagataaga

tttcatttgaaatcattttgaggaatcattggtctataaaactcttttatagaaaaacat

agggggtatagctcagctggtagagcgttgtccttgcacggcaaatgccagcggttcgag

tccgcttacctccacaaacccaaagagagagatatacatagtcttatgtatactcattta

actctaaatgcgtaaccaatcgtatttttgggctactagctcagttggttagagcgtacc

cctgataagggtaaggtctcaggttcaaatcctgagtagcccaaaccggcttgctttctt

gcttgcaggtattgaattgatagaaaataaacttaacaaatatttttatttgaaaaagtt

aaagactaaaaaaaagacattgataaagaaacaaagggcttatggcggatccctaggcac

tcagagacgatgaaaggcgtgtttaccgacgaaacgcttcagggagctggaagagtgcat

tgatctgaagattc

>FN563095 *Mamiella gilva* Chlorophyta 1406f/23Sr

cacaccatggaagtcgactcttccctaagttgttactcgaaccctctgggacgaggatgc

cgaaggcagggttggtgactggggtgaagtcgtaacaaggtagccgtactggaaggtgtg

gctggaatacctccttttaaagatgtaacttttcggcgccgcaactcgcagtatgctgag

atgcaggcgccaagcgctacgggctatgagctcacagcatgtacatgctgggcgctacag

cgcctagacgtgtatgggctattagctcaggtggttagagcgcacccctgataagggtga

ggtctcaggttcaagtcctgaatagcccatggcacgggggtatagctcagttggtagagc

gctgcctttgcaaggcagacgccagcggttcgagtccgcttacctccagccaaccacgca

ttgaacgtggaaatgaattgtggtattttgagtttgaaaaaaatcaatcaataaagggcg

tatggtggagacctagggacctagagccgatgaagggcgtgacaaccgacgaaatgcctc

ggggagcaggacgtatgcgtcgatccgaggattc

>FN563096 *Mantoniella squamata* Chlorophyta 1406f/23Sr

cacaccatggaagtcgactcttccctaagtcgttactcgaacccattgggacgaggatgc

cgaaggcagggttggtgactggggtgaagtcgtaacaaggtagccgtactggaaggtgtg

gctggaatacctcctttttaaagatgaataaagcgcgcattttgtcctgcatgaggcttg

ccttatgaaagcgatgaatgcaccacgctacgggctattagctcaggtggttagagcgca

cccctgataagggtgaggtctctggttcaagtccagaatagcccatggcacgggggtata

gctcagttggtagagcgctgcctttgcaaggcagacgccagcggttcgagtccgcttacc

tccagccaagccttttcacccaagctttgcttgagtgcatcaaggcatatcagaattgcg

gttatttcgatacacaaaacaaaatcaatcaataaagggcatatggtggagacctaggga

cctagagccgatgaagggcgtgacaaccgacgatatgcctcggggagcaggacgtatgca

ctgatccgaggattc

>FN563097 *Micromonas pusilla* Chlorophyta 1406f/23Sr

cacaccatggaagtcgactcttccctaagtcgttactcgaacccattgggacgaggatgc

cgaaggcagggttggtgactggggtgaagtcgtaacaaggtagccgtactggaaggtgtg

gctggaatacctcctttttaaagatgaattaagcgcgcatttttcaacctagttggcttg

ccactaaaatatgaaaaatgcaccacgctacgggctattagctcaggtggttagagcgca

cccctgataagggtgaggtctctggttcaagtccagaatagcccatggcacgggggtata

gctcagttggtagagcgctgcctttgcaaggcagacgccagcggttcgagtccgcttacc

tccagccaagcctaagaaagcttcttaggcatatcagaattgcggttatttgaatacaca

aaacaaaatcaatcaataaagggcatatggtggagacctagggacctagagccgatgaag

ggcgtgacaaccgacgatatgcctcggggagcaggacgtatgcactgatccgaggattc

>FN563098 *Micromonas pusilla* Chlorophyta 1406f/23Sr

cacaccatggaagccgactcttccctaagtcgttactcgaacccattgggacgaggatgc

cgaaggcagggttggtgactggggtgaagtcgtaacaaggtagccgtactggaaggtgtg

gctggaatacctcctttttaaagatgaaacaagcgcgcatttcttaaaacaattggcttg

ccattgcttgagaagaaatgcaccacgctacgggctattagctcaggtggttagagcgca

cccctgataagggtgaggtctcaggttcaagtcctgaatagcccatggcacgggggtata

gctcagttggtagagcgctgcctttgcaaggcagacgccagcggttcgagtccgcttacc

tccagccaaacctttaatttaaaaggtcaattagaattgtggttatttcgacacataaaa

caaaatcaatcaataaagagcatatggtggagacctagggacctagagccgatgaagggc

gtgacaaccgacgatatgcctcggggagcaggacgtatgctttaatccgaggattc

>FN563101 *Pyramimonas disomata* Chlorophyta 1406f/23Sr

cacaccatgggagctggccatgcccgaagttgttaccctaaccgtgaggagggggatgcc

taaggtagggctagtgactagggtgaagtcgtaacaaggtagccgtactggaaggtgcgg

ctggatcacctcctttatcaaggtttagaaaccaaaaatagcgttgggctattagctcag

ctggtaagagcgcacccctgataagggtgaggactctggttcgaatccagaatagcccaa

cccaatgaattctcatggaagatgggggtatagctcagttggtagagcgctgcctttgca

aggcagatgtcagcggttcgagtccgcttacctccatcgttcggtgcacgcacctgaacc

atgaaataaatcaaaatgaattaagggcttacggtggatacctaggcacctagagacgat

gaaaggcgtagtaaccgacgaaacgcttcggggagctggaaacaagctttgatccggaga

ttc

>HE610156 *Trichosarcina mucosa* Chlorophyta 1406f/23Sr

cacaccatggaagctgattgggcccgaagtcgttgtaaacgcctaaggcacagttagtga

ctggggtgaagtcgtaacaaggtagnnnnnctggaaggtggccctggatcacctccttca

aaaatagaaaaagcttttgttccgtgataagccttatatctgcttagactccctgtccgt

ttctggacaagcgcgtcttcgaagcccctgaatacgtgtcttcgaagagaagcgcgttta

ggtctggtttcgcggaaacaaagtaaagcttactagtcatacaatcagaattacggtgtg

cgcgtttaaaaccgcgaccgccgcttttctgagggctattagctcagttggttagagcgc

gcccctgataagggcgaggtcactggttcaaatccagtatagcccacccatttaagagaa

aagtggggttatagctcagttggtagagcgctgtctttgcacggcagatgtcagcggttc

gaatccgcttaactccacatgtcgcagatacccttagcccagaatatttatccgtgcaac

tattttaaaagaagtggttgtaaagtttttgttctgagcacaaaattaggatttctgtct

caaagctgctttctaaacttttccgtaggtcgtacgtaggtcgggctcttttacgggtcc

tgtcaaagcaatcttcgacgacaaagtaaccaaaaactttgccggtcattgtatgtttgt

cattgtatgtctgtcaaagacacctctgcgaacacctttgcgaaggactttgtacctgcg

gttaggctagaaaccgcttttttttcttaaagttttggtcaaatatattaaggcttacgg

tggatacctaggcattcagagacgatgaagggcgtggataccgacgaaacgcttcgggga

gttggaaacaagcattgatccgaagattc

>HE610157 *Oltmannsiellopsis sp. CCMP1240* Chlorophyta 1406f/23Sr

cacaccatggaagttggttccgcccgaagtcgtggatctaaccttttggaagaaagcgcc

tacggtgtaactagtaactatggtgaagtcgtaacaaggtatccgtactggaaggtgtgg

attggaatacctcctttaaaggacaaattaagactaaatttagtcatttaaaagaatcaa

atgtgtattggtaattctttaccaatacgcaaccgattacttatataaagtccacgcgga

cttcatatgaagtccgcatcaggtgggtatatagctcagttggtagagcgctgtctttgc

acggcagatgtcagcggttcgaatccgcttatatccacctgaaatatcgagtcgggctat

tagctcagttggttagagcgcgcccctgataagggcgaggtcgctggttcaaatccagca

tagcccactcgaacaaatacgcagacbcggcgcaatcttgcgccgagtcattgcggtcaa

atgacaataggcttatggtgaatacctaggcacccaaagttgatgaagggcgcggaaacc

ggcgaaacgcttcggggagttggaaacacgctttgatccgaagatac

>HE610167 *Pedinomonas sp. UTEX 'LB 1027'* Chlorophyta 1406f/23Sr

cacaccatgggagctggctatgcccaaagtcgttaccccaaccgtttggagggggatgcc

taaggcagagctagtgactggggtgaagtcgtaacaaggtagccgtactggaaggtgcgg

ctggatcacctcctttttaaggtccattcaaaattttcaagaaacaacttgatgaagtca

cgggctattagctcaggtggttagagcgcacccctgataagggtgaggtcgctggttcga

gtccagcatagcccatgactccgcaatgttgaagttgaaggacactgttcagcaagcata

ttgctgatcaaagatccaaagccggtttgtgaccgggggtatagctcagttggtagagcg

ctgcctttgcaaggcagatgtcagcggttcgagtccgcttacctccaccaaatcgaaaaa

cttttggatcaagcatttttttgttaagttaaaacttagcaataaacatatttcttttaa

ttttaagaaattaaaattaaacctgcttatgttaaagcataaggtcaaaggaaatacgga

ttacggtggatacctaggcatctagagtcgaagaagggcgtcgaaaccaacgaaaagctt

cgggaagctggaaacaagctatgatccggagattc

>HE610168 *Pedinomonas tuberculata* Chlorophyta 1406f/23Sr

cacaccatgggagctggctatgcccaaagtcgttaccctaaccgcaaggagggggatgcc

taaggcagagctagtgactggggtgaagtcgtaacaaggtagccgtactggaaggtgcgg

ctggatcacctcctttttaaggtccttaaaaagacttacactcaaaaagtggaagacaaa

acttctacgtaaacagtagaaagtaaaacttatacttaaaaagtgtaaagtccttaggct

tttcagccaaagctggaaaggtccttgaaactttcaattctgaaagtttttactatctca

acatcttgagaaaaaaagtcaaaagtgttctgctaccaagcagggcacgggctattagct

caggtggttagagcgcacccctgataagggtgaggtcgctggttcgagtccagcatagcc

catgacttaaaatcaaagatcttcgagatagaaaatcacaaaatcggtttgtgccagtgc

ttatcgctttgctgcaagcatgcgatgtgcctgggggtatagctcagttggtagagcgct

gcctttgcaaggcagatgtcagcggttcgagtccgcttacctccaccaaatcgatacaga

ttttggatccacacaaactttgtttgtgtaaataaaatctaggaattatactcctaacca

gcaaggcacgtcagcttcgttgttactctgctaacgagcaaagcgcgtcagcttcgttgt

ttaacgtataattttcccagatctagtttctcaaaactaaaaagtaattctatctttttc

aagatagaaaaacaaatacggtcaaaggaaatacagattacggtggatacctaggcatct

agagtcgaagaagggcgtcgaaaccaacgaaaagcttcggggaagctgtaaacaagcaaa

gctggaaacaagctatgatccggagattc

>HE610169 *Pedinomonas sp. M2079/1* Chlorophyta 1406f/23Sr

cacaccatgggagctggctatgcccaaagtcgttaccctaacctttggagggggatgcct

aaggcagagctagtgactggggtgaagtcgtaacaaggtagccgtactggaaggtgcggc

tggatcacctcctttttaaggatcttacaagctttctcttttgaaaaagcttttactcga

gaatcgtcgtcaaaaagtcacgggctattagctcaggtggttagagcgcacccctgataa

gggtgaggtcgctggttcgagtccagcatagcccatgacttcaaatcaagacgcgattct

tagaaaatcacatcaacgatttgagaacgggggtatagctcagttggtagagcgctgcct

ttgcaaggcagatgtcagcggttcgagtccgcttacctccaccaaatcggaaattgattt

tggatcagccgaaactcgcaagaggatcgcaagattcctcgcaagagaatctttttaact

tttactcgaaagaggtcaaaggaattacggattacggtggatacctaggcatctagagtc

gaagaagggcgtcgaaaccaacgaaaagcttcgggaagctggaaacaagctatgatccgg

agatac

>HQ700713 *Schizomeris leibleinii* Chlorophyta 1406f/23Sr

caaaggtcggaaggagatagggtcggaagtggtttcgcttaacgatgaaaatcgagggca

gctacaaattctctgtttccaactaatcttaagtcgtaacaaggtagccctactggaagg

tggggctggaggactccttcttaaaataaaaaagtattaatatacttcgtactttctttt

ttaaagaaagtaaccatctttttaatatttaaaagatggagggcaactttttaataaagt

tgcacagcattcagcaaagacatcttgctgggatagggctattagctcagttggttagag

cgcacccctgataagggtgaggtcactggttcaagtccagtatagcccaccagttggtct

tttataaaacttttttcccaaaagattctttggaaatactgtttaggtggggatatagct

cagttggtagagcgctgcctttgcaaggcagatgccagcggttcgaatccgcttatctcc

accctttttaaaaaggattccattcgatttttttttggtgaaaacaaaactgttttaaat

tgtgtgttgttacaaggtttatactaaaaagaacctctaaaggtcaaatgaagtaaggcg

gacggcggagacctaggcactcagagacgatgaagggcgcataaaccggcgatacgcttc

ggggagctggaaacgagctttgatccgaagattc

>ED495993 *Sorghum bicolor* Streptophyta ITSF/ITSFReub

ggggaacctgcggttggatcacctccttaccttaaagaagcgtactttgcagtgctcaca

cagattgtctgatgaaaatgagcagtaaaacctctacaggcttgtagctcaggtggttag

agcgcacccctgataagggtgaggtcggtggttcaagtccactcaggcctaccaaatttg

cacggcaaatttgaagaggttttaactacatgttatggggctatagctcagctgggagag

cgcctgctttgcacgcaggaggtctgcggttcgatcccgcatagctccaccatctctgta

gtggttaaataaaaaatacttcagagtgtacctgcaaaggttcactgcgaagttttgctc

tttaaaaatctggatcaagctgaaaattgaaacactgaacaatgaaagttgttcgtgagt

ctctcaaattttcgcaacacgatgatggatcgcaagaaacatcttcgggttgtgaggtta

agcgactaagcgtacac

>EI077474 *Oryza nivara* Streptophyta ITSF/ITSFReub

ggggaacctgcggttggatcacctccttaccttaaagaagcgtactttgtagtgctcaca

cagattgtctgatagaaagtgaaaagcaaggcgtttacgcgttgggagtgaggctgaaga

gaataaggccgttcgctttctattaatgaaagctcaccctacacgaaaatatcacgcaac

gcgtgataagcaattttcgtgtccccttcgtctagaggcccaggacaccgccctttcacg

gcggtaacaggggttcgaatcccctaggggacgccacttgctggtttgtgagtgaaagtc

gccgaccttaatatctcaaaactcatcttcgggtgatgtttgagatatttgctctttaaa

aatctggatcaagctgaaaattgaaacactgaacaacgagagttgttcgtgagtctctca

aattttcgcaacacgatgatgaatcgaaagaaacatcttcgggttgtgaggttaagcgac

taagcgtacac

>FJ789639 *Brassica rapa* Streptophyta ITSF/ITSFReub

ggggaacctgcggctggatcacctcctttctaaggatcgtgacgaaagcgtcagtgctag

acactgaaagagcttcgtcatttccaaagaacatagccgccgtcctcatgtcccttcatc

actagagattagcgcagttcgctgcgctgatagctgagcaggctcaagcgcctctggctg

ctaacgcagcctgatttggcagctgggccggtagctcaggtggttagagcgcacgcctga

taagcgtgaggtcggaggttcaactcctccccggcccaccagcatttggtgaggggcttt

agctcagctgggagagcggttgctttgcaagcatcaggtcatcggttcgatcccgataag

ctccaccatttgctgcgcaaatggtcttttgatttgggtccgctgcataagcagcgacgg

ccgttcggccttgcgaaccttcggttcgttggagcgccaatcaagcgatctgccagatac

tctagttgatgaagatagcggtttaccagccaaggctggtgatatggctcgcacaagcga

gcctctttgacattgtgaatgggttttttaatcgatgccgtggcgacatggttcggtttt

tggtgtttccgcaagggagcatccggaaagcgggcgatgttgtacacacaagattatctg

gctgagtttaataaccacaccgatacagctttcgacaaatgctacccagtattgtcgttg

gtggtgtggactctcaagcgtgaggtaagggcatct

>GU799579 *Carica papaya* Streptophyta ITSF/ITSFReub

ggggaacctgcggttggatcacctccttaccttaaagaagcgttctttgcagtgctcaca

cagattgtctgatagaaagtgaaaagcaaggcgtttacgcgttgggagtgaggctgaaga

gaataaggccgttcgctttctattaatgaaagctcaccctacacgaaaatatcacgcaac

gcgtgataagcaattttcgtgtccccttcgtctagaggcccaggacaccgccctttcacg

gcggtaacaggggttcgaatcccctaggggacgccacttgctggtttgtgagtgaaagtc

acctgccttaatatctcaaaactcatcttcgggtgatgtttgagatatttgctctttaaa

aatctggatcaagctgaaaattgaaacactgaacaacgaaagttgttcgtgagtctctca

aattttcgcaacacgatgatgaatcgaaagaaacatcttcgggttgtgaggttaagcgac

taagcgtacac

>AM084273 *Paulinella chromatophora* None ITSF/ITSFReub

ccggaaggtgcggctggatcacctcctaacagggagacaaaatgtgttactaatgtttgg

ctacttatattgccattctggtatcctgtcaccttaggtcgatcggtatcttatttcgta

agtctggattttgtaattcagctatcatttcagttcctaaactttgtctaagtcatgccc

cggcaagggtctcctgggccattagctcaggtggttagagcgcacccctgataagggtga

ggtccctggttcaagtccaggatggcccattcgtgtttgggggtttagctcagttggtag

agcgcctgctttgcaagcaggatgtcagcggttcaaatccgctaacctccattgaccaaa

ttcttctaaatcaagaaggaagatctttgaagtagcatgagactagacacctaagctgaa

ttagaattcagcatttttcttacttagactggtcaaactaaaagtaaaaccagtctaagt

aagaaaaatgctgaactccttgtaatgagactaaatcttagttttcattagagatagttc

ggccgaaccttgacaactacataggcaaaattagaagaataagtttcttatgggggctta

cctaaataatagtagtccatttgttagactattgctataaggataagcttctagccagaa

taaaatttgatgttttgatataaatgcctgcttttaatttgattaaaacaggccaaaatc

atgatattagtgaatctattttgatgatctgccctcgtgattttgaagtttatcaaaaaa

ttaatctggttaagctacaaagagctcac

>CP000815 *Paulinella chromatophora* None ITSF/ITSFReub

ccggaaggtgcggctggatcacctcctaacagggagacaaaatgtgttactaatgtttgg

ctacttatattgccattctggtatcctgtcaccttaggtcgatcggtatcttatttcgta

agtctggattttgtaattcagctatcatttcagttcctaaactttgtctaagtcatgccc

cggcaagggtctcctgggccattagctcaggtggttagagcgcacccctgataagggtga

ggtccctggttcaagtccaggatggcccattcgtgtttgggggtttagctcagttggtag

agcgcctgctttgcaagcaggatgtcagcggttcaaatccgctaacctccattgaccaaa

ttcttctaaatcaagaaggaagatctttgaagtagcatgagactagacacctaagctgaa

ttagaattcagcatttttcttacttagactggtcaaactaaaagtaaaaccagtctaagt

aagaaaaatgctgaactccttgtaatgagactaaatcttagttttcattagagatagttc

ggccgaaccttgacaactacataggcaaaattagaagaataagtttcttatgggggctta

cctaaataatagtagtccatttgttagactattgctataaggataagcttctagccagaa

taaaatttgatgttttgatataaatgcctgcttttaatttgattaaaacaggccaaaatc

atgatattagtgaatctattttgatgatctgccctcgtgattttgaagtttatcaaaaaa

ttaatctggttaagctacaaagagctcac

>CP000815 *Paulinella chromatophora* None ITSF/ITSFReub

ccggaaggtgcggctggatcacctcctaacagggagacaaaatgtgttactaatgtttgg

ctacttatattgccattctggtatcctgtcaccttaggtcgatcggtatcttatttcgta

agtctggattttgtaattcagctatcatttcagttcctaaactttgtctaagtcatgccc

cggcaagggtctcctgggccattagctcaggtggttagagcgcacccctgataagggtga

ggtccctggttcaagtccaggatggcccattcgtgtttgggggtttagctcagttggtag

agcgcctgctttgcaagcaggatgtcagcggttcaaatccgctaacctccattgaccaaa

ttcttctaaatcaagaaggaagatctttgaagtagcatgagactagacacctaagctgaa

ttagaattcagcatttttcttacttagactggtcaaactaaaagtaaaaccagtctaagt

aagaaaaatgctgaactccttgtaatgagactaaatcttagttttcattagagatagttc

ggccgaaccttgacaactacataggcaaaattagaagaataagtttcttatgggggctta

cctaaataatagtagtccatttgttagactattgctataaggataagcttctagccagaa

taaaatttgatgttttgatataaatgcctgcttttaatttgattaaaacaggccaaaatc

atgatattagtgaatctattttgatgatctgccctcgtgattttgaagtttatcaaaaaa

ttaatctggttaagctacaaagagctcac

>DQ369902 *Zea mays* Streptophyta ITSF/ITSFReub

ggggaacctgcggctggatcacctccttaatcgacgacatcagctgctccataagttccc

acacgaattgcttgattcattgaagaagacgataaagaagcagcccgaaattgggtctgt

agctcagttggttagagcgcacccctgataagggtgaggtcggcagttcgaatctgccca

gacccaccaattttgtgtgggaaacgcctgtagaaatacggggccatagctcagctggga

gagcgcctgccttgcacgcaggaggtcaacggttcgatcccgtttggctccaccattaac

tgtttctactgttagagtttagaaatgaatattcgccaatgaatattgatttctagtctt

tgattagatcgttctttaaaaatttgggtatgtgatagaaagatagactgaacgttactt

tcactggtaacggatcaggctaaggtaaaatttgtaagtaattgcgaattttcggcgaat

gtcgtcttcacagtataaccagattgcttggggttatatggtcaagtgaagaagcgcata

c

>DQ369903 *Zea mays* Streptophyta ITSF/ITSFReub

ggggaacctgcggttggatcacctccttaccttaaagaacctgcctttgtagtgctcaca

cagattgtctgatgaaaaacagcagtaaaaatctctgcaggcttgtagctcaggtggtta

gagcgcacccctgataagggtgaggtcggtggttcaagtccactcaggcctaccagactc

cttttaaaggatgagcggtacagagatatataaacgatggggctatagctcagctgggag

agcgcctgctttgcacgcaggaggtctgcggttcgatcccgcatagctccaccatctttt

actgcgaacacaagaaaacttcagagtgaacctgaaaaggtgcactgcgaagttttgctc

tttaaaaatctggatcaagctgaaaattgaaacgacacatcttaatggtgtgttcgagtc

tctcaaattttcgcaatcagaagtgaaacatcttcgggttgtgaggttaagcgactaagc

gtacac

>DQ369905 *Zea perennis* Streptophyta ITSF/ITSFReub

tcggaaggtgcggctggatcacctcctttctaaggaaaacgtcccttacgggacatgccc

atcgttcagttttgagagctcgtctctcagtctcgatagagacactcgcaccttgaaaac

tgaagacatcaacaagacatcaaactttttataaccatgtcattagacgtgtgttcttag

aataccaaaatgctagatcaaggtatgaagggcgtac

>DQ369907 *Zea diploperennis* Streptophyta ITSF/ITSFReub

ggggaacctgcggttggatcacctccttacctgaagataccttcccgcgcagtgctcaca

cagattgtctgataaaaagtaatgagcaagacggctgcgaagtcgtgacactacccgtgt

ccccttcgtctagcggttaggactccgccctttcacggcggcaacaggggttcgaatccc

ctaggggacgccacttgcttggtgacaggtgaaaggtgtctctacgaagtatctcaaaac

tgacttaaccgtcatgtttgagatattgctctttaacaatccggaacaagctgaaaattg

aaacgacgtgttggttcatttctccgtaataggaaatgaataacaacatgttcgagtctc

tcaaatgcttgcagtccgcagcgttgcaaaacgcctgtgggttgtgaggttaagcgacta

agcgtacac

>DQ369909 *Zea mays* Streptophyta ITSF/ITSFReub

ggggaacctgcggttggatcacctccttaccttaaagaacctgcctttgcagtgctcaca

cagattgtctgatgaatgatgaacttctgaatgtacttttgagtgcattaagaagttttg

ctctttaaaaatctggatcaagctgaaaattgaaacgacacatctttaatggtgtgttcg

agtctctcaaattttcgcaatcagaagtgaaacatcttcgggttgtgaggttaagcgact

aagcgtacac

>DQ369911 *Zea mays* Streptophyta ITSF/ITSFReub

ggggaactgcggttggatcacctccttacctaaaagatacaaacccgcgtagtgctcaca

cagattgtctgatagaaaacgagcagtaaaaccttataggcttgtagctcaggtggttag

agcgcacccctgataagggtgaggtcggtggttcaagtccactcaggcctaccaaatttt

cccctgttctgcgttgcacctcagactcgcatacttaagtatgcgtcgctaagttgcgcc

ttgaccagatgaaaattcatggtaatcaaaggttttacgaaatcgatggggctatagctc

agctgggagagcgcctgccttgcacgcaggaggtcagcggttcgatcccgcttagctcca

ccatcatttcatgcaccaaaacttacttcagagtgtaccggcgacggtgtactgcgaagt

atttgctctttaacaatccggaacaagctgaaaattgaaacgacatgtcgtctcattcct

ccgtaataaggaatggggttaagacatgttcgagtctctcaaattttcgcaacagcgatg

gtgtctcacgagacatcttcgggttgtgaggttaagcgactaagcgtacac

>DQ369913 *Zea mays* Streptophyta ITSF/ITSFReub

ggggaacctgcggctggatcacctccttaatcgacgacatcagctgctccataagttccc

acacgaattgcttgattcattgaagaagacgatagaagcagctttaagctccaagctgat

agctcttagctaatcagttacgcgctcgaaattgggtctgtagctcagttggttagagcg

cacccctgataagggtgaggtcggcagttcgaatctgcccagacccaccaattttgttat

ggggccatagctcagctgggagagcgcctgccttgcacgcaggaggtcaacggttcgatc

ccgtttggctccaccattaactgtttctactgttagagtttagaaatgaatattcgccaa

tgaatattgatttctagtctttgattagatcgttctttaaaaatttgggtatgtgataga

aagatagactgaacgttactttcactggtaacggatcaggctaaggtaaaatttgtaagt

aattgcgaattttcggcgaatgtcgtcttcacagtataaccagattgcttggggttatat

ggtcaagtgaagaagcgcatac

>HF562234 *Chondrus crispus* None ITSF/ITSFReub

ctggaaggtgcggctggatcacctccttaattagggaaaatagttatacaacaattctat

taaccttagatcgtatattaaataatttttaagggctattagctcagatggttagagcgc

acccctgataagggtgaagtccctggttcaattccaggatagcccaaatgaaatagtagg

ggggtatagctcagctggtagagcgctgcctttgcaaggcagatgtcagcggttcgagtc

cgcttatctccacaaagttaagttgtacttctcaaataaaattttattatttgataataa

aaaaaagtaaagtaagtaagagcttac

>JF810595 *Nitella hyalina* Streptophyta ITSF/ITSFReub

ggggaacctgcggctggattgactcctttccaatatacccaaccagctcttccttccttc

cttcgagcctctgtgaatgtcgatcgtctcatcagactagtatcaatcatacaaaggcac

aaaataacatgaagatcatgaaccagaaaaagacccgatccaggcacggcacatccggcg

tcgtcacgaacgcatgcacgcacgcgtgcaattgaatgaagaggcacgaagtgcaacaca

acaaatgatgaaaaaaaagcgaaggaggcacaattccgagtacttttccgggtactattg

taggacgtgaaaacacccgatcccattccgacctcgatccgtgaaatcgtcttacgccat

atgtactgatttttgcattttgggagacatggttcaagcccggaatgtgcctttgataaa

tagtagtgtattgactctggcgcacttcgtgcctggttgctcttacaatgtcggtcgcct

tatcaagtccattcgaagctttgcggtttgatttgactccaggcacattcatagaggctc

gccagagcagacgcgaagcgtctatcgccagagttgacgtggaagggccaggctgggcag

gttccataatacgcgggatagagtaattggtaactcgtcaggctcataatctgaatgttg

taggttcaaatcctactcccgccaaaggctcgccagagtgggcgaaaacggatggacaaa

acaatctcgtactattcgtatttgtttttttagaagatccaaagaaaagaagtatcttct

ataagtttgtgtggtcattttgggtatttctgataaggcgaccgacatacatttgtagag

gcaaccacgccctgacgggccttaggctctaagagtcgggatataaacaaaaagcaagtt

agaagtagggctgcgccctgctatatccaaaataatgcattg

>KC894740 *Grateloupia taiwanensis* None ITSF/ITSFReub

ctggaaggtgtggctggatcacctccttaattagggtttgcaaagtgaacttaataactt

tacatcccagatcgaaaatataagagctcttaatttaagtgttctaagaaatttgagtaa

ctaagggctattagctcagttggttagagcgcacccctgataagggtgaggtccctggtt

caaatccaggatagcccagctcaaagtagcgggggtatagctcagttggtagagcgctgc

ctttgcaaggcagacgtcagcggttcgagtccgcttatctccaaaaaaataattcaacta

tttaagcttagactaactaagcttaaatagaatgtactagtatatgttagtacaagtatt

aagtaaatttaaggcttac

>X54299 *Antithamnion sp.* None ITSF/ITSFReub

ctggaaggtgcggctggatcacctccttatttagggatatactagattaatttataataa

tcttatcctaggtcgtaattttcgggctattagctcagttggttagagcgcacccctgat

aagggtgaggtccctggttcaaatccaggatggcccaagcaaaagggggtatagctcagt

tggtagagcgctgcctttgcaaggcagatgtcagcggttcgagtccgcttatctccacgc

aaaatagtaaaataaatttactaaatataatactaaaattaattataaattaatttttaa

attttatttatagttttatgtataataaaatatcaagtcattaagggcttac

>Z29521 *Chondrus crispus* None ITSF/ITSFReub

ctggaaggtgcggctggatcacctccttaattagggaaaatagttatacaacaattctat

taaccttagatcgtatattaaataatttttaagggctattagctcagatggttagagcgc

acccctgataagggtgaagtccctggttcaattccaggatagcccaaatgaaatagtagg

ggggtatagctcagctggtagagcgctgcctttgcaaggcagatgtcagcggttcgagtc

cgcttatctccacaaagttaagttgtacttctcaaataaaattttattatttgataataa

aaaaaagtaaagtaagtaagagcttac
